# The profile of imported malaria in Sri Lanka from 2013 to 2023

**DOI:** 10.1186/s44263-026-00242-5

**Published:** 2026-01-14

**Authors:** Pubudu Chulasiri, Deepika Fernando, Shilanthi Seneviratne, Rajitha Wickremasinghe, Champa Aluthweera, Thilan Fernando, Kumudu Gunasekera, Kamini Mendis

**Affiliations:** 1 Anti Malaria Campaign, Ministry of Health, Colombo, Sri Lanka; 2 Department of Parasitology, Faculty of Medicine, University of Colombo, Colombo, Sri Lanka; 3 Department of Public Health, Faculty of Medicine, University of Kelaniya, Ragama, Sri Lanka

**Keywords:** Imported malaria; Sri Lanka, Delayed diagnosis, Chemoprophylaxis, Labor migration, Travelers’ malaria

## Abstract

**Background:**

Sri Lanka was certified malaria-free by the World Health Organization in 2016 and has remained so since then. Yet, imported malaria cases and the presence of mosquito vectors in parts of the country threaten the re-establishment of malaria.

**Methods:**

Data on imported malaria cases diagnosed from 2013 to 2023 in Sri Lanka were extracted from the National Malaria Database containing detailed data on every case of malaria maintained at the Anti Malaria Campaign, Sri Lanka. Descriptive analyses were carried out to characterize imported malaria in the country.

**Results:**

A total of 532 imported malaria cases were reported during the study period, over half of them (68.5%) were those who traveled for employment as low-wage or high-wage workers. Infections with all four human malaria species were imported, with a majority being *Plasmodium (P.) falciparum* (48.1%), most acquired in Africa, and *P. vivax* (40.5%), most acquired in India. Imported *P. ovale* infections took longer to manifest clinically from time of travel (median 95 days) than did other Plasmodia infections (median 14 days). Infections with *P. malariae* took longer to diagnose from onset of illness (median 15.5 days) than other *Plasmodium* species (median 4**–**6 days). Patients accessed healthcare for their malaria illness sooner (geometric mean = 2.42 days) than physicians took to diagnose malaria (geometric mean 3.41 days) (*p* < 0.001). Seven patients with *P. falciparum* recrudesced and three with *P. vivax* relapsed. Forty-six imported malaria cases were severe, all but one due to *P. falciparum*, and one death occurred among them over the study period. Epidemiological features of imported malaria were species-specific and related to the biology of the *Plasmodium* species.

**Conclusions:**

A large proportion of imported malaria, predominantly *P. falciparum* and *P. vivax*, was associated with work-related travel. The parasite species incidence over the years followed trends of incidence reported from Africa and India from where they were predominantly imported. The delay in diagnosing imported malaria which increases the risk of morbidity and mortality, and also risks the re-establishment of malaria in the country were mainly on the part of the physicians and not due to patients delaying seeking treatment.

**Supplementary Information:**

The online version contains supplementary material available at 10.1186/s44263-026-00242-5.

## Background 

Sri Lanka was one of twenty-one countries which eliminated malaria and were certified by the World Health Organization (WHO) since 2000. Among them, Sri Lanka, Maldives, China, El Salvador, Belize, Carbo Verde, Egypt, Suriname, and Timor Leste are either entirely or in part in the tropical belt [1]. Thus, the malaria-free zone now extends to the more tropical regions of the world. Until recently, imported malaria was being reported mostly from temperate countries, in travelers to the tropics who are either tourists or expatriates returning from visiting family and friends in their countries of origin [2, 3]. However, lately, with many countries in the tropics being freed of malaria, the profile of imported malaria has changed radically, reflecting a high degree of south-to-south travel associated with labor rather than leisure-related travel [4–7].

In the past when most imported malaria cases were being reported from temperate countries, its significance was the association of travelers’ malaria with high morbidity and mortality due to delays in diagnosis, malaria being a rare disease in the reporting country. Now, in malaria-free tropical countries that are highly receptive to the disease because of the high densities of the mosquito vector, imported malaria carries an additional risk of re-establishing malaria in that country. This has major implications for countries to sustain their malaria-free status.

Sri Lanka, which achieved malaria-free status in 2016, has high densities of malaria vectors in parts of the country, and an ecological environment which is conducive to malaria transmission. The principal malaria vector in Sri Lanka is *Anopheles culicifacies*, and there are several other secondary vectors as well. Introduction of the parasite through imported infections risks the re-establishment of malaria with potentially devastating consequences in a now, non-immune population [8]. Thus, with over 50 imported malaria cases being reported annually in Sri Lanka, the country’s objectives are twofold—one to prevent the re-establishment of malaria and two to sustain a zero malaria mortality in travelers [9]. This study reviews malaria imported to Sri Lanka over a period of 11 years following the elimination of the disease in 2012, to profile imported malaria characteristic of the tropics with its newer associated risk of labor migration. It provides a basis for strategies to both reduce malaria mortality in travelers and prevent the re-establishment of malaria.

## Methods

The Strengthening the Reporting of Observational Studies in Epidemiology (STROBE) checklist for cross-sectional studies [10] is provided in Supplementary Material 1: Table S1.

Data from 2013 to 2023 were extracted from the national malaria database maintained at the Anti Malaria Campaign (AMC), Sri Lanka, which records detailed information on every reported malaria infection in the country, all being imported except for two. The AMC is a specialized campaign within the Ministry of Health Sri Lanka which has been mandated to control, eliminate, and now prevent the re-establishment of malaria. It is staffed by community health physicians, medical officers, entomologists, and parasitologists and other technical and administrative staff. A case of malaria is classified as imported based on a recent travel history to a malaria endemic country, and particularly in cases where the interval since travel is longer than a few weeks, on the absence of any evidence of local transmission in the areas of residence of the patient. All malaria cases reported in the country are reviewed monthly by the independent case review committee of experts comprising a malariologist, parasitologist, epidemiologist, pharmacologist, and a clinician in addition to invited staff of the AMC [11]. A case is classified as imported, indigenous, relapse, recrudescence, or transfusion-induced based on WHO guidelines after verification of findings and stringent review of the Case Review Committee; the findings of the case review committee are recorded in the national register and these data are used for statistical purposes.

On each patient, a detailed history including personal data, history of travel, past malaria infections, dates of the current infection, and health seeking pattern; and clinical records including laboratory findings, clinical management and treatment outcomes, and follow-up data were recorded. Case surveillance comprises both passive case detection in the public and private health sectors, and active case detection by way of proactive surveillance by screening high-risk groups entering the country, and reactive surveillance when a malaria case is detected [12]. Initial diagnosis is based on both microscopy and rapid diagnostic tests (RDTs), and when required using polymerase chain reaction (PCR). Every case is confirmed by microscopy. Quality-assured microscopy is performed based on the standard operating procedures [13]. Confirmatory microscopy was performed by WHO accredited expert malaria microscopists at the Central Laboratory of the AMC. Routinely, the thick blood smear is examined for identification of the stages of malaria parasites (asexual stages and gametocytes), and details of microscopic procedures are described in the standard operating procedures of the AMC [13]. Data on infections with the four main species of human malaria parasites, namely, *Plasmodium vivax*, *Plasmodium falciparum*, *Plasmodium ovale*, and *Plasmodium malariae*, were compared. Data on some of the variables analyzed, i.e., information regarding diagnosis and chemoprophylaxis, were only available from 2019 to 2023. Records with regard to the hemoglobin (Hb) level and platelet count at the time of diagnosis, which are measured as a part of a full blood count assessment via an automated hematology analyzer (Mindray BC-6800Plus, Shenzhen, China), were available over a 4-year period from 2020 to 2023. A patient was classified as having severe malaria as defined by the WHO [14, 15].

Methods used in diagnosis and management of imported malaria have been described elsewhere [16–19]. Time to diagnosis is defined in this paper as the time taken from the onset of symptoms to obtaining a confirmed laboratory diagnosis of malaria. Patients were treated in accordance with the national malaria treatment guidelines [18, 20]: *P. vivax* with chloroquine and primaquine, the latter after G6PD testing, and uncomplicated *P. falciparum* with oral artemisinin-based combination therapy (ACT), artemether- lumefantrine (AL), and a single dose of primaquine. G6PD testing was carried out whenever possible using a quantitative spectrophotometric analysis which measures the precise levels of the G6PD enzyme in red blood cells to determine the severity of any deficiency. In the absence of advanced laboratory facilities, the qualitative Brewer’s test was carried out. Severe malaria was treated with intravenous (IV) artesunate followed by a complete course of oral ACT as directly observed treatment [18]. Case investigation was carried out for every patient diagnosed with malaria [21] as has been previously described [16, 17, 19, 22, 23].

Following treatment, patients were followed up both clinically and parasitologically for 3 days (D0**–**D2) in hospital as in-ward patients, and thereafter on days 7, 14, 21, and 28. In *P. falciparum* infections, patients were followed up on day 42 to detect recrudescences.

*P. vivax* and *P. ovale* malaria patients were followed up for relapses for a further 12 months with monthly blood smears [17, 18].

If a recurrent infection was diagnosed in an individual previously diagnosed with malaria and received radical treatment, re-infection was ruled out by the absence of a travel history to a malaria endemic country and extensive entomological and parasitological reactive case detection which was carried out to exclude any local transmission. Recrudescence in *P. falciparum* was confirmed by genotyping the polymorphic regions from *P. falciparum* merozoite surface proteins (msp1 and msp2) and glutamate-rich protein (glurp) coding sequences of both the initial and the recurrent samples [24].

Prophylactic antimalarial medicines, namely, mefloquine or doxycycline, for prophylaxis against *P. falciparum* for individuals traveling to Africa, and chloroquine for those traveling to India and other countries in Asia are provided free-of-charge [25]. From 2019 onwards, the history of using chemoprophylaxis and adherence to the regimens was recorded in all patients.

Data were analyzed using Microsoft Excel 365 (Microsoft Corporation Inc., Redmond, WA, USA, Version 2019) and IBM SPSS Statistics version 21 (SPSS Inc., IBM Corp., Armonk, NY, USA, Version 21). Descriptive statistics were used to summarize demographic, epidemiological, and clinical characteristics. Categorical variables are presented as frequencies and percentages. Continuous variables are presented as medians with interquartile ranges, while skewed time-interval variables were summarized using geometric means.

## Results

### Species composition and country of origin of imported malaria

**Fig. 1:**
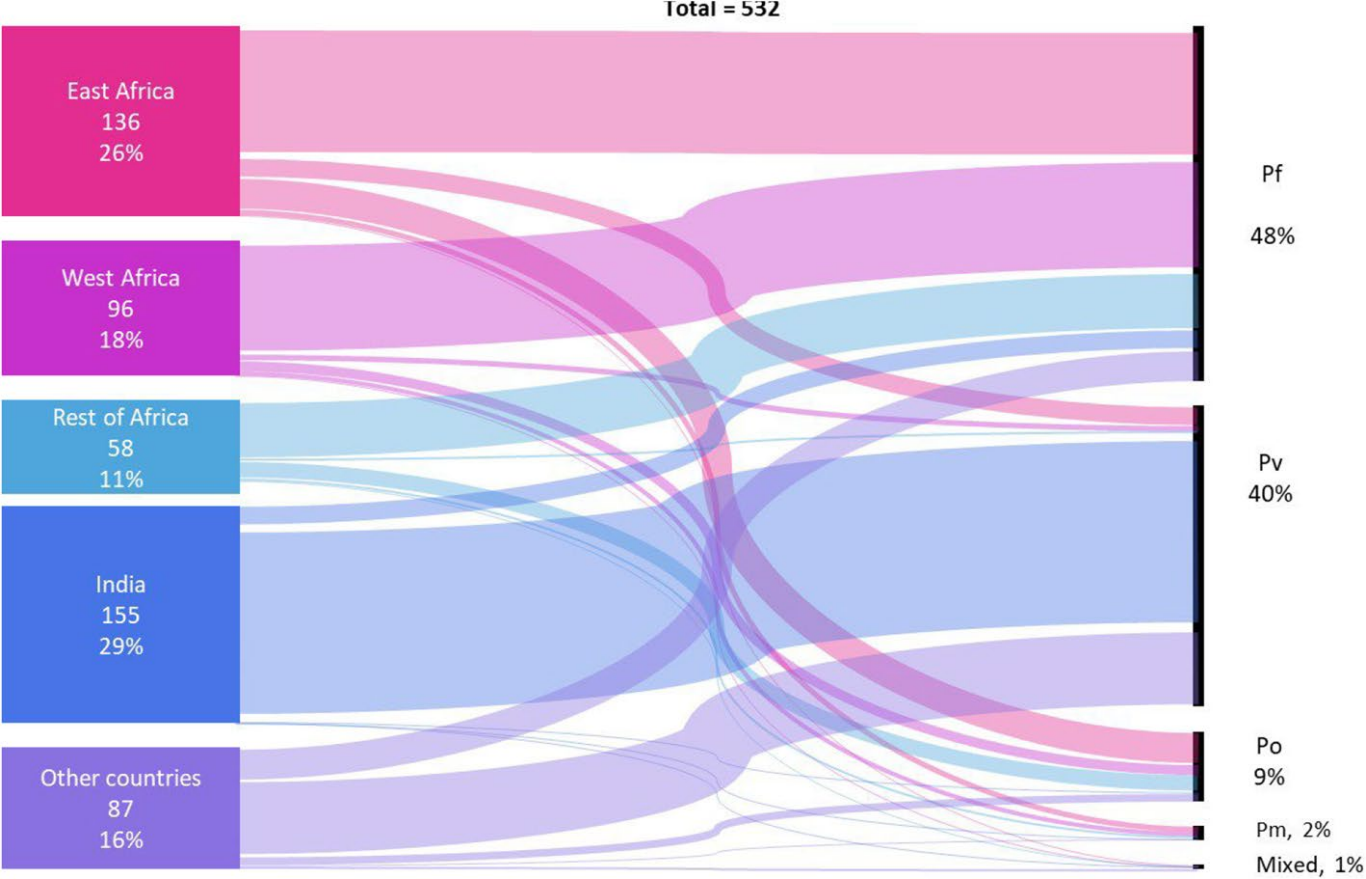
Geographical origin of imported malaria species-wise. Legend: Geographical regions and countries from where malaria was contracted are indicated on the left with numbers and percentages of imported malaria cases. On the right are shown the parasite species as a percentage of all imported cases

Over the 11-year period 2013**–**2023, 532 imported malaria cases were reported in Sri Lanka. Of these, 256 (48.1%) were due to *P. falciparum* (including two mixed infections; one with *P. ovale* and the other with *P. malariae*), 215 (40.5%) due to *P. vivax*, and the rest due to *P. ovale* (9.4%), *P. malariae* (1.9%), and *Plasmodium knowlesi* (0.19%). A majority of these infections originated from Africa (54.5%; *n* = 290) and India (29.1%; *n* = 155). Seventy-two percent of infections originating from African countries were *P. falciparum*, while 90% of those originating from the Indian subcontinent were *P. vivax*. With the exception of two *P. ovale* infections which originated in China and India respectively, the rest of the *P. ovale* infections and all the *P. malariae* infections were acquired in Africa (Fig. 1; Supplementary Material 2: Table S2). The only case of *P. knowlesi* was acquired in Malaysia [26].

One hundred and forty of the 215 *P. vivax* infections (65%) were acquired in India (Supplementary Material 2: Table S2); 88 (63%) of these infections were diagnosed in Sri Lankan nationals, 47 (34%) in Indian nationals, and the rest in other foreign nationals arriving from India. The rest 15% of *P. vivax* infections were acquired in Africa. Of the *P. falciparum* infections, 231 (90.2%) were acquired in Africa, the highest proportion of infections being acquired in East Africa (40.7%). Of the *P. falciparum* infections originating in Africa, 197 (85.3%) were in Sri Lankan nationals and 34 (14.7%) in foreign nationals. The rest of the 25 *P. falciparum* infections were acquired in other countries, including 14 (5.5%) from India (Supplementary Material 2: Table S2 and Fig. 1). The age and gender distribution of imported malaria infection are given in Supplementary Material 2: Table S3.

**Fig. 2:**
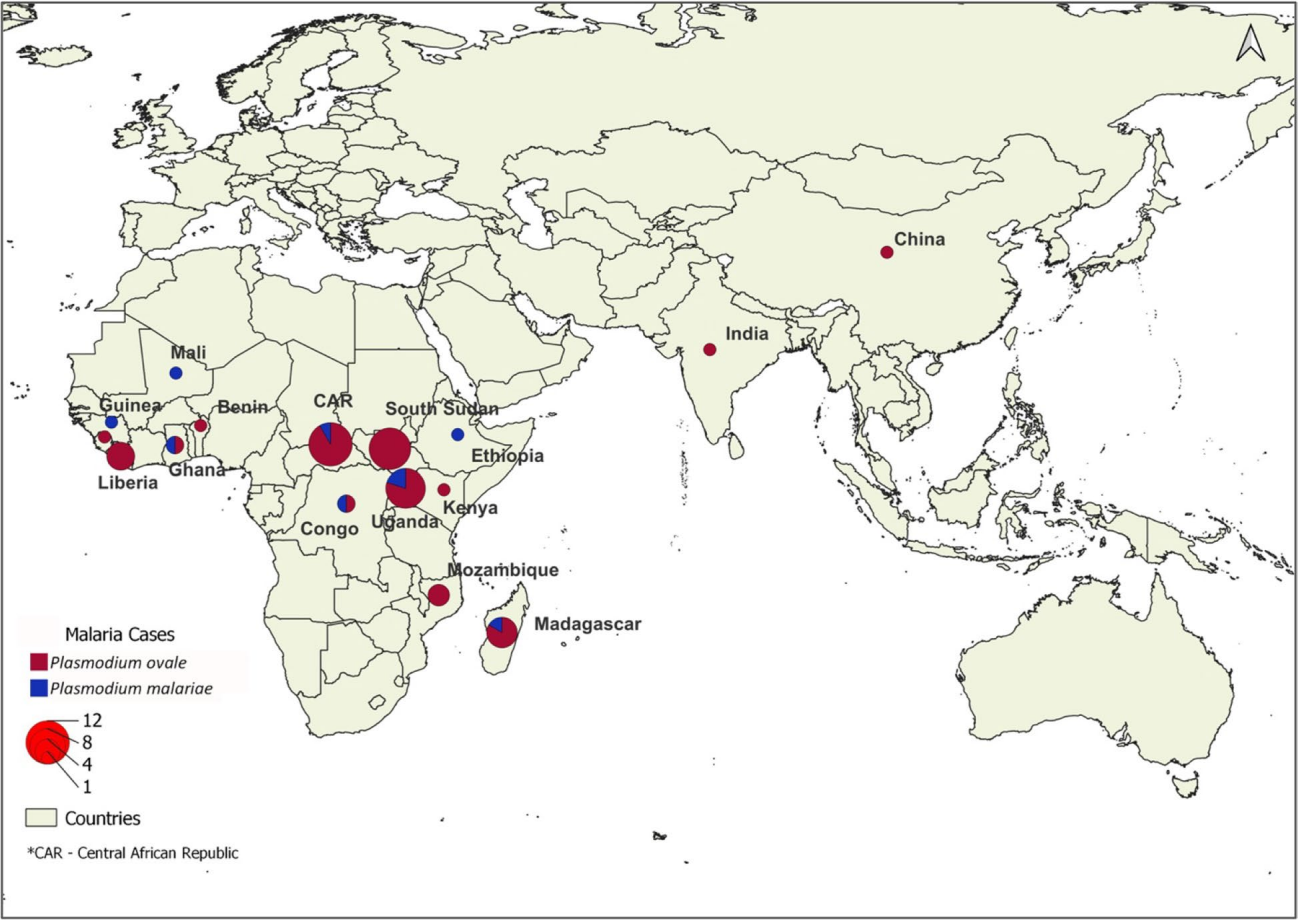
Geographical origin of imported *P. ovale* and *P. malariae* malaria. Legend: Map showing the countries of origin of imported *P. ovale* (red) and *P. malariae* (blue) cases. The diameter of the circles represents the upper limit of the number of cases and the colors indicate the proportion of each species from that location

Approximately 90% of all *P. ovale* and *P. malariae* infections were diagnosed in Sri Lankan nationals (46/50 *P. ovale* and 9/10 *P. malariae*). Forty-eight cases of *P. ovale* and all cases of *P. malariae* originated from Africa; the highest number of *P. ovale* (*n* = 58.3%) cases originated in East Africa while *P. malariae* cases originated equally from East and West Africa (Fig. 2).

**Fig. 3:**
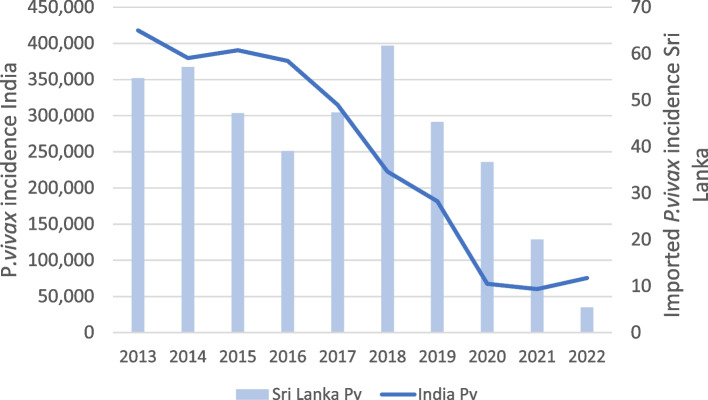
Incidence of *P. vivax* reported from India and of imported *P. vivax* in Sri Lanka. Legend: The line represents the number of reported *P. vivax* cases in India during the study period [26], and columns represent the number of imported *P. vivax* cases in Sri Lanka during the same period

**Fig. 4:**
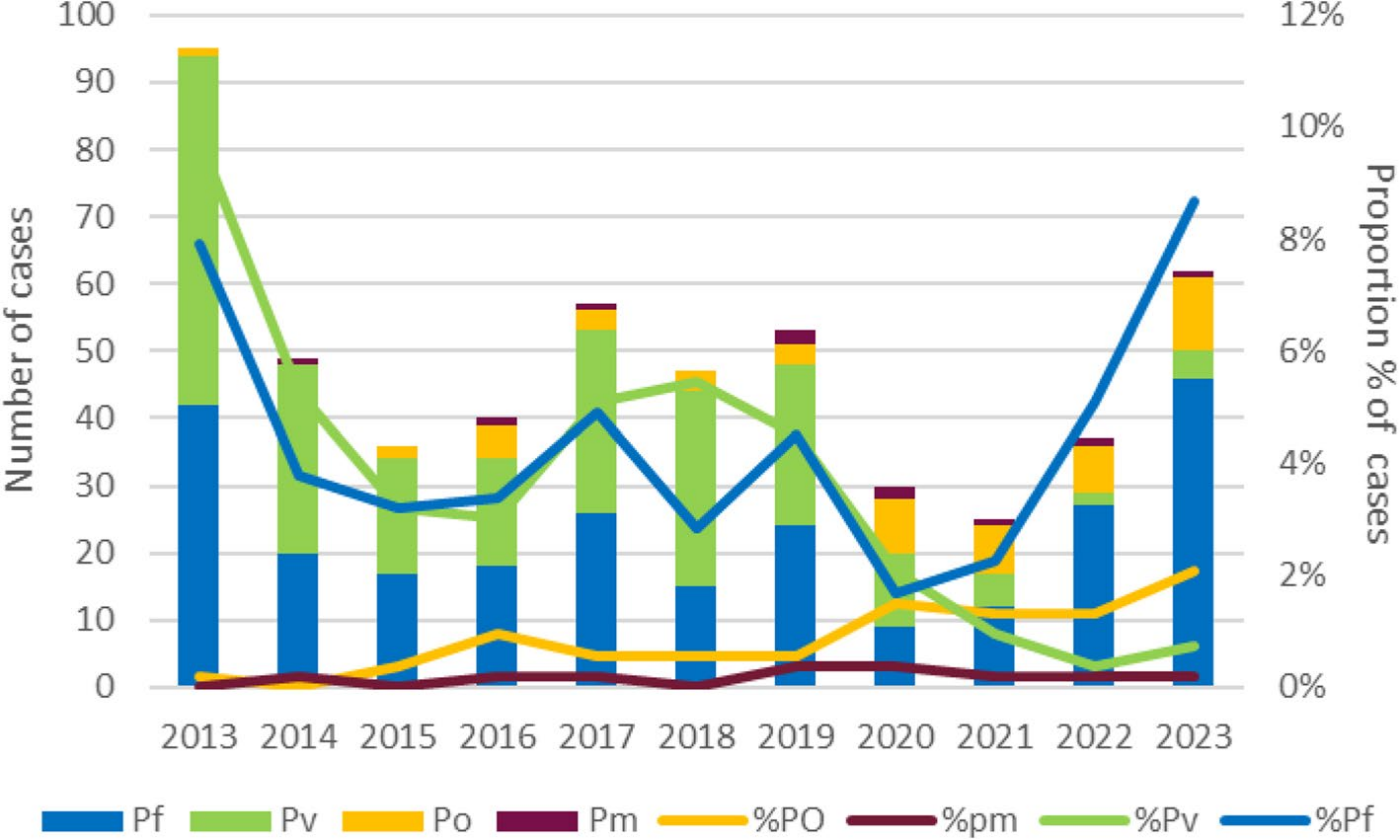
Imported malaria cases in Sri Lanka by year and species. Legend: The number of imported malaria cases shown by stacked columns by species: *P. falciparum* in blue; *P. vivax* in green; *P. ovale* in orange; and *P. malariae* in red. And the relative proportions of each species shown by lines of the corresponding colors

Over time, the proportion of *P. falciparum* cases increased steadily from 30 to 74.19% in 2023. In Sub-Saharan Africa which is the main source of imported *P. falciparum* infections, there were some reductions in incidence and mortality beginning in 2000, but after 2019 progress stalled [27]. In contrast, the proportion of imported vivax malaria infections in the same period declined from 54.7% in 2013 to 6.45% in 2023, the decrease being steeper after 2017. India, the main source of imported *P. vivax* infections has seen a steady and steep decline in malaria [27] (Fig. 3). The proportions of both *P. ovale* and *P. malariae* cases also showed an increasing trend since 2014 (Fig. 4).

**Fig. 5:**
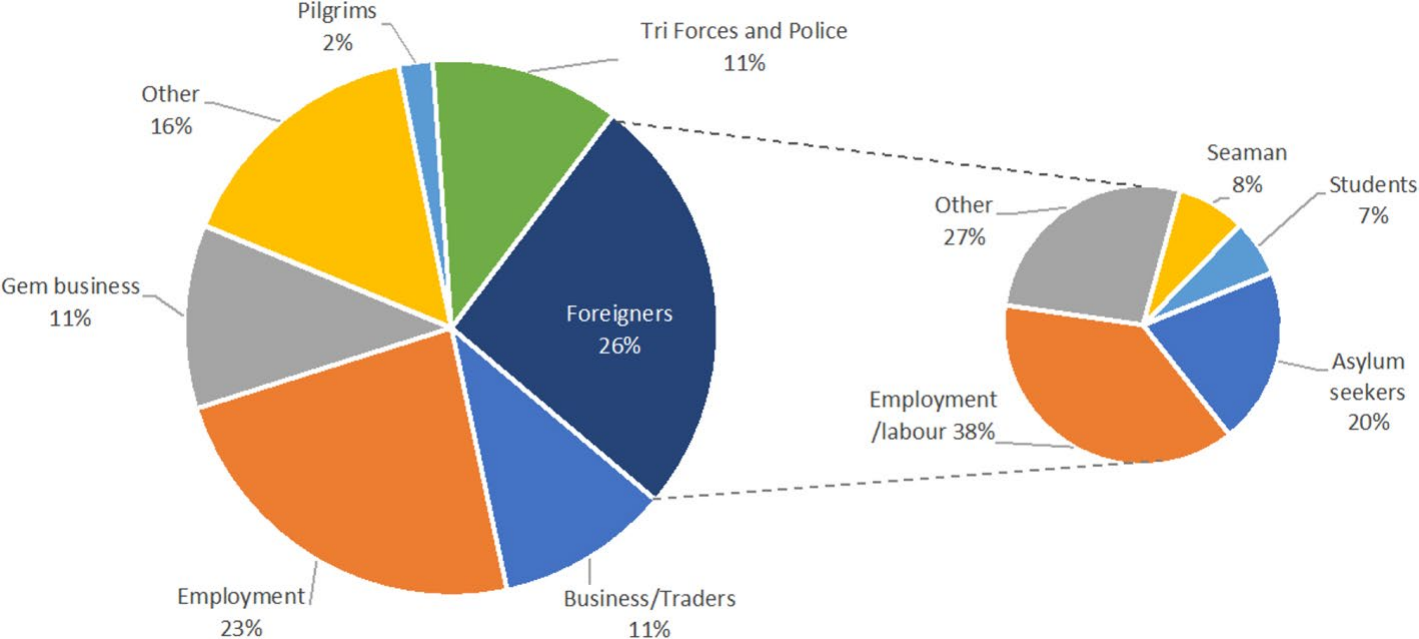
Purpose of international travel of imported malaria patients among Sri Lankan nationals and foreign citizens. Legend: The pie chart on the left shows the proportions (%) of imported malaria patients based on the purpose of international travel of Sri Lankan nationals and the proportion of foreign nationals. The chart on the right shows the proportion (%) of imported cases among foreign nationals based on the purpose of travel

Of all imported malaria infections, the highest percentage (68.5%; *n* = 364) was among people who traveled for employment in or from malaria endemic countries which included both low-wage (e.g., physical labor, fisherman) and high-wage workers (technical grades such as electricians, plumbers, carpenters). Sixty-one (11%) were tri-forces personnel who returned from United Nations (UN) Peacekeeping missions in Africa; many had spent approximately 1 year overseas. Fifty-nine (11%) were gem traders also returning from Africa but spending only a few days to months during each visit. Amongst the 137 foreign nationals who were diagnosed with malaria, 52 (38%) were migrant workers, 28 (20%) were asylum seekers, and the rest 37 (27%) were mostly tourists (Fig. 5).

### Time from arrival in the country to onset of symptoms

*P. ovale* patients took the longest to manifest clinical disease after arrival in the country, the median duration between their arrival in Sri Lanka, and the onset of illness being 95 days (IQR 172.25 days). Patients with infections of other species presented with clinical disease sooner after arrival in the country, the median duration being 14 days (IQR 82.75 days) in *P. vivax*, 14.5 days (IQR 160.72 days) in *P. malariae*, and 3 days (IQR 8.0 days) in *P. falciparum* (Table 1).

**Table 1:** Duration between arrival in Sri Lanka and onset of symptoms^1^ (2013–2023)

Duration between arrival in Sri Lanka and onset of symptoms	Number of cases (%)^2^			
	*P. ovale* (*n* = 50)	*P. malariae* (*n* = 10)	Uncomplicated *P. falciparum* (*n* = 207)	Uncomplicated *P. vivax* (*n* = 205)
< 15 days	12 (24%)	5 (50%)	183 (88%)	101 (50%)
15 days**–**1 month (30 days)	4 (8%)	1 (10%)	13 (6%)	21 (10%)
1**–**3 months (31**–**90 days)	8 (16%)	1 (10%)	4 (2%)	37 (18%)
3**–**6 months (91**–**180 days)	13 (26%)	1 (10%)	3 (1%)	23 (11%)
> 6 months (> 181 days)	13 (26%)	2 (20%)	4 (2%)	23 (11%)
**Median (IQR) (days)**	95 (172.25)	14.5 (160.72	3 (8.0)	14 (82.75)

^1^Only uncomplicated mono-infections were considered

^2^Percentages calculated column-wise. There were some missing data

### Time to diagnosis from onset of symptoms

This information was available in 476 patients; the longest time to diagnosis from onset of symptoms was in *P. malariae* patients, the median duration being 15.5 days (IQR 60.25 days), and it being 4**–**6 days in infections with the other species (Table 2).

**Table 2:** Time to malaria diagnosis following onset of symptoms^1^ (2013–2023)

**Time to malaria diagnosis following onset of illness**	*P. ovale* (*n* = 50)	*P. malariae* (*n* = 10)	Uncomplicated *P. falciparum*^2^ (*n* = 207)	Uncomplicated *P. vivax*^2^ (*n* = 205)
< 8 days	35 (70%)	3 (30%)	150 (72.1%)	131 (63%)
8 or more	15 (30%)	7 (70%)	58 (27.9%)	77 (37%)
**Median (IQR) (days)**	5 (3.0)	15.5 (60.25)	4 (6.0)	6 (7.0)

^1^Only uncomplicated mono-infections were considered

^2^ Some data were missing

**Fig. 6:**
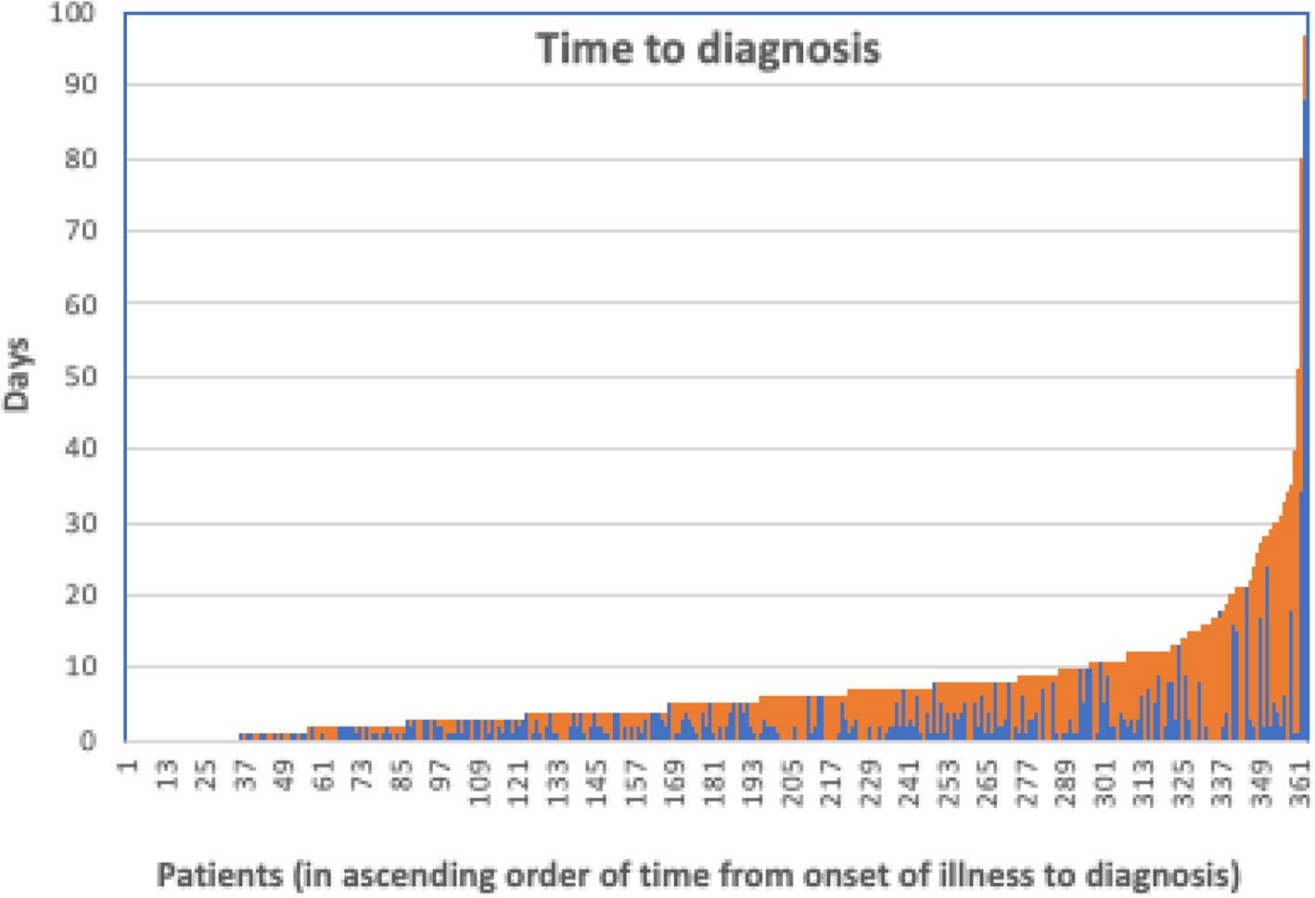
Time to malaria diagnosis following onset of symptoms. Legend: The time to a malaria diagnosis of patients shown by columns, from the onset of symptoms to their first contact with the health system (in blue) and from their first contact with the health system to diagnosis (in orange). Patients are arranged in ascending order of total time taken from onset of illness to diagnosis of malaria (complete data was available in only 361 patients)

The time to diagnosis is a composite of (a) the time taken by the patient from onset of symptoms to access healthcare (i.e., seek treatment) and (b) the time taken by the health system to diagnose malaria, i.e., from the patients’ first contact with a healthcare provider to a confirmed malaria diagnosis. These two time periods were therefore analyzed separately. Patients took on average 2.42 days (geometric mean) to access healthcare for their malaria illness and physicians took on average 3.41 days (geometric mean) to diagnose malaria from the time they first saw the patient, the two periods being significantly different (*p* =  < 0.001) (Table 2, Fig. 6).

### Presence of infectious stages

Of those diagnosed with non-falciparum malaria species, 84% had gametocytes, the infective states of the parasite, in addition to asexual stages in the peripheral blood. In contrast, 19% of patients with *P. falciparum* infection had gametocytes at the time of diagnosis.

### Clinical features and treatment outcomes

All individuals diagnosed with malaria were admitted to either a government (72.2%) or a private hospital (27.8%) and treated in-ward for a minimum duration of 3 days.

The mean Hb levels in infections with the four species *P. ovale*, *P. malariae*, *P. vivax*, and *P. falciparum* on day 0 were 12.69 g/dl, 10.12 g/dl, 11.72 g/dl, and 13.03 g/dl, respectively. Thrombocytopenia was a feature of infections with all four species but neither the Hb levels nor the degree of thrombocytopenia were significantly different between the four *Plasmodium* species.

All *P. vivax* patients recovered with treatment but three patients relapsed despite radical treatment of the primary infection; the duration between the confirmed primary infections and the relapse were 2.4, 6.4, and 10 months. These three relapses were detected when the patients presented with a new clinical episode and not during routine follow-up. These three infections were classified as relapses based on the fact that there was no history of travel to a malaria endemic country between the primary and recurrent infection and also based on the results of entomological surveillance and parasitological reactive case detection which excluded any possibility of local transmission in the area. There were no relapses detected amongst any of the *P. ovale* patients during the follow-up period.

Amongst the 256 *P. falciparum* infections diagnosed, seven (2.7%) had a recurrent infection within the 28 days of follow-up which were confirmed as therapeutic failure of the first-line medicine and they were all treated successfully with the second-line medicine.

*P. ovale* and *P. malariae* patients had uncomplicated clinical disease and made a complete recovery.

### Severe malaria and deaths

Over the 11-year study period, 46 severe imported malaria infections were reported, of which 45 were due to *P. falciparum*, and one due to *P. vivax*. The severe *P. vivax* malaria infection was in a traveler who returned from Madagascar who had co-infections with HIV and tuberculosis. In one severe *P. falciparum* patient who returned from Africa in 2023, the disease was fatal and this case has been described elsewhere [19].

### Compliance with chemoprophylaxis

Data on the use of chemoprophylaxis was available only for Sri Lankan nationals and only from 2019 to 2023. A third of the patients had obtained prophylaxis which is provided free-of-charge by the AMC prior to travel. None of these patients who obtained prophylactic medicine adhered to the drug schedule as advised by the AMC (Table 3). Continuation of the medicines, following their return to Sri Lanka, i.e., mefloquine, or chloroquine (weekly for 4 weeks after return), were also poorly adhered to. Between 2019 and 2023, the proportion of Sri Lankan nationals who developed *P. ovale* infections while on chemoprophylaxis (mefloquine) was higher (76%) than that of any other species (19**–**34%). Based on data from 2020 onwards, the cost to the AMC of providing mefloquine as chemoprophylaxis to the tri-forces personnel alone was approximately 8700 USD per year.

**Table 3:** Adherence to prescribed chemoprophylaxis amongst Sri Lankan nationals stratified by *Plasmodium* species detected (2019–2023)

**Adherence to chemoprophylaxis**	*P. ovale* (*n* = 34) n (%)	*P. malariae* (*n* = 6) n (%)	*P. vivax* (*n* = 36) n (%)	*P. falciparum* (*n* = 99) n (%)	Total (*n* = 175) n (%)
Yes, irregular	26 (76%)	2 (33.3%)	7 (19.4%)	25 (25.3%)	60 (34.3%)
No	8 (24%)	4 (66.7%)	29 (80.5%)	74 (74.7%)	115 (65.8%)

## Discussion

The principal malaria vector in Sri Lanka has been *Anopheles culicifacies*, there being several other secondary vectors as well. It is predominantly a rural mosquito present mainly in the dry zone which occupies about 2/3rds of the country. Its continued presence in the country constitutes a risk for re-establishing malaria transmission from imported malaria cases. In order to prevent the re-establishment of malaria, the Anti Malaria Campaign uses two main strategies: (1) the early detection and prompt treatment of imported malaria patients using active and passive case surveillance to reduce morbidity and mortality and prevent the onward spread of the parasite, and (2) mosquito vector surveillance and control of vectors in situations where the risk of transmission is high. Effective case surveillance depends on knowing the risk profile of imported malaria patients so that interventions can be targeted. A successful program to prevent the re-establishment of malaria also requires recognizing the characteristics of imported malaria disease so that the risk of onward transmission and morbidity and mortality of imported malaria can be reduced.

Almost three-fourths of individuals importing malaria to the country were Sri Lankan nationals. Among all imported malaria patients, 68% were those who traveled for employment, and 23% of them did so as low-wage labor. The rest of work-related patients included members of UN Peacekeeping missions returning from Africa (11%) and gem traders (11%). Foreign nationals who imported malaria were fewer, largely migrant workers, both legal and illegal, mainly from the Indian sub-continent, and asylum seekers from Asian countries. Tourists accounted for about 16% of cases among foreign nationals. Travelers for employment constitute a large and important risk group because the health seeking behavior, particularly of low-wage labor, tends to be restrictive for early detection of malaria [28]. Sri Lanka makes considerable investments to provide chemoprophylaxis to prevent malaria in, and follow up, security forces returning from missions overseas [29–31]. The same is true of a large community of gem traders in a coastal belt of the country whose members travel frequently between Madagascar, India, and Sri Lanka and constitute a risk category which is more difficult to track and follow up because they travel singly or in small groups and for short durations of time. This data shows that the population profile of imported malaria has shifted from one associated with leisure travel such as tourists and expatriates returning from visiting friends and family in the past as reported from temperate countries [2], to work-related travel of both low- and high-wage labor in tropical countries that have eliminated malaria. This has implications for health systems, because people of the lower social and economic stratum such as migrant labor tend to have more risky health-seeking behaviors, e.g., delay in seeking treatment for illness, and complying less with health advice [28]. Therefore, efforts have to be made to provide access to preventive and curative services within the public sector. These changes also call for active case detection in high-risk population to be promoted for malaria diagnosis rather than merely depending on patients seeking treatment on their own. Sri Lanka has adopted a migrant health policy [32] within which access to malaria diagnosis and treatment is provided free-of-charge within the public sector to any Sri Lankan or foreign national. This is an incentive specially for foreign labor to seek testing and healthcare for illness and has improved case detection and clinical outcomes of imported malaria.

Most imported *P. falciparum*, *P. ovale*, and *P. malaria*e cases originated from Africa, and most *P. vivax* cases originated in India. The species profile of imported malaria will depend on the volume of travel to the various travel destinations over time, and on the intensity of transmission in those countries as reflected in the data presented here. There is no evidence that travel patterns to these different regions changed much over the period of this study except during the COVID pandemic when all international travel was curtailed [33]. Therefore, the incidence trends of imported malaria species over the past 11 years closely followed the trends in the burden of malaria in the African continent and India.

Almost 10% of imported malaria cases were *P. ovale* and much less *P. malariae*. With the exception of two cases of *P. ovale* which originated in outside Africa, the rest originated from African countries; the relative proportions of these two species are consistent with their proportions as reported from several regions including Sub-Saharan Africa [27, 34–37]. Importation of both species showed a slight increasing trend over time. Other countries which have neared elimination or have eliminated malaria have also reported an increasing trend in imported *P. ovale* cases lately. In Anhui Province in China, which has not reported any autochthonous cases of malaria since 2014, the proportion of imported malaria caused by *P. ovale* species increased significantly over the period 2021**–**2023 [38]. As these data show, the trends of imported malaria in malaria-free areas and countries will reflect the malaria burden in endemic areas and countries over time provided that travel patterns remain unchanged. By the same reasoning, imported malaria in non-endemic countries may serve as a sampling pool for transmission trends and other aspects of malaria such as the prevalence and emergence of drug-resistant parasites in endemic countries.

In most imported *P. falciparum* infections, the clinical symptoms manifested within 2 weeks of the person arriving in the country, while in infections with the other species, the interval from travel to the onset of clinical disease was longer and varied widely. Twenty percent of patients with *P. ovale* and *P. malariae* became clinically ill beyond 6 months after arrival in the country. The time to clinical patency was longest in *P. ovale* (median = 95 days). These differences may be partially accounted for by differences in the biology of the parasite species. *P. falciparum* has a shorter incubation period (6**–**14 days) than other species, in *P. vivax* it being 12**–**17 days, *P. ovale* 12**–**20 days, and *P. malariae* 18**–**40 days [39, 40]. *P. vivax* and *P. ovale* are relapsing parasites and some of the imported infections seen here could well have been relapses following a primary infection which occurred elsewhere. Relapses may account for the longer interval between the person’s arrival in the country and the first episode of malaria in *P. vivax* and the very long interval in *P. ovale* as observed in this study. Other studies have also reported *P. ovale* malaria as manifesting long after the patients’ arrival. In a long-term study, conducted between 1987 and 2015 on imported malaria cases in the UK, the median “incubation” period of *P. ovale* was 76 days [41]; in a retrospective study in Sweden between 1995 and 2019, it ranged from 27 to 653 days after arrival of the traveler in the country [42].

The use of blood stage chemoprophylactic agents (i.e., mefloquine, doxycycline, or chloroquine), as was the case in some of the patients in this study, will also delay the time to clinical patency. These preventive medications do not act against hypnozoites; therefore, they will only prevent a blood infection for the duration that preventive treatment is taken and not any further, and relapses could therefore account for delays in the appearance of clinical disease particularly in *P. vivax* and *P. ovale* [43]. The longer the period since the time of a person’s travel that a malaria infection takes to clinically manifest, the more likely it is that the history of travel will be forgotten or ignored. A history of travel being an important cue for the physician to suspect and test for malaria delayed appearance of illness therefore increases the risk of a diagnosis being delayed. Imported *P. ovale* malaria may, therefore, be particularly at risk of a delayed diagnosis because it took on average 95 days to manifest clinical symptoms after arrival in the country.

Delays in the diagnosis of imported malaria are one of the major challenges faced by malaria-free countries because it increases the risk of mortality in travelers’ malaria; in countries where the vector is present, it also increases the risk of re-establishing malaria transmission [8]. Over the 11-year period following malaria elimination in the country, there was, in fact, a single occurrence of an onward mosquito-borne transmission from an imported infection to a resident of Sri Lanka, i.e., an “introduced” case of malaria; any further transmission from this infection was prevented by mounting a swift and effective response [16] but this event was a testimony to the risk of onward transmission from an imported infection and of re-establishing malaria in the country. In the present study, the median number of days to diagnose malaria infections ranged from 4 to 6 days from the onset of illness in all *Plasmodium* species except in *P. malariae* in which it was longer, 15.5 days. Evidence has been previously presented that a delay in diagnosis of imported *P. falciparum* malaria of more than 5 days places patients at a significantly higher risk of developing life-threatening severe malaria [44]. Mortality due to travelers’ malaria is often assumed to be due to delays in patients seeking treatment. The data presented here show that to the contrary, patients seek treatment within a relatively shorter time than physicians take to arrive at a diagnosis of malaria. Delays in diagnosing imported malaria are, therefore, mostly on the part of the health system rather than on the part of the patient. Malaria rapid diagnostic tests and quality assured microscopy for malaria are widely available throughout Sri Lanka, and a 24-h hotline operated by the AMC provides advice to physicians and the public when a malaria patient is suspected. A major effort is being made to increase the awareness of travelers and well as physicians of the risk of imported malaria [45–47]. Yet, it is the rarity of malaria in the country (30**–**60 imported malaria cases a year) and a high incidence of other microbial diseases causing fever that makes clinicians repeatedly test for dengue, leptospirosis, and other viral diseases before suspecting and testing for malaria. This has resulted in a high morbidity in imported malaria patients. One death in a *P. falciparum* malaria patient mentioned here and published earlier [19] and another in 2024 (after the period covered by this study, therefore data not shown) raises the case fatality rate of imported *P. falciparum* malaria in Sri Lanka to 0.67% which is at the high end of the range reported globally. Therefore, efforts to increase the awareness of both travelers and physicians on the need for malaria testing must be a continuing process, even more so in countries that are at risk of malaria re-establishment. Previous studies have assessed the effectiveness of existing diagnostic strategies for imported malaria in Sri Lanka in an effort to avoid delays in diagnosis [12, 48].

Data presented here reveals that antimalarial chemoprophylaxis was either not obtained by most patients (66%) or not adhered to despite efforts being made by the AMC to increase traveler awareness, and providing medicines free-of-charge to overseas travelers. Troops proceeding on UN peacekeeping missions to Africa are issued with sufficient amounts of mefloquine as prophylaxis free-of-charge at the cost to the AMC of approximately 8700 USD per year. Poor adherence of travelers to chemoprophylaxis was mainly due to unpleasant adverse effects and forgetting to take the medicines as prescribed [29, 30]. Given the poor adherence, the cost-effectiveness of this intervention may need to be assessed. Tri-forces personnel are also provided with long-lasting insecticidal bed nets with advice on how to test for malaria should they develop fever while overseas and after their return. Similarly, gem traders, a clearly identified high-risk group visiting Africa, are also regularly provided information on malaria preventive measures through advocacy campaigns and even house visits. These measures, and continuously monitoring the time to diagnosis in every imported malaria patient, have been important components of the current prevention of re-establishment of malaria program in Sri Lanka. The data presented here show that a surprisingly high proportion of travelers who developed *P. ovale* infections were on mefloquine prophylaxis, although their degree of compliance was unknown and even questionable. Other studies have also reported that chemoprophylaxis seems to fail more frequently with *P. ovale* [49, 50]. Nolder et al. stated that the proportion of *P. ovale* malaria which occurred in patients reporting chemoprophylaxis use (33%) was significantly higher compared to that in *P. falciparum* (6.4%) and *P. vivax* (23.7%) [51]. Similarly in a study carried out in France, between January 2006 and December 2017, amongst the imported malaria infections diagnosed, 47% of individuals who developed *P. ovale* had taken chemoprophylaxis as compared to 30% of individuals who developed *P. vivax* and had used chemoprophylaxis [43]. It is likely that most of these *P. ovale* infections are relapses, which as mentioned above become patent long after the chemoprophylactic effect has ceased [43].

It is not possible to discern if imported *P. vivax* and *P. ovale* infections which are relapsing parasite species were primary infections or relapses because the history of past episodes of malaria while abroad were unreliable and most patients had no documented evidence of past malaria episodes. However, three out of 215 imported *P. vivax* patients relapsed after arrival giving a relapse rate of 1.4%, and none of *P. ovale* patients relapsed during the study period, after their previous infection was detected and treated with primaquine in Sri Lanka.

Seven patients with *P. falciparum* infections originating in Africa recrudesced within the 28-day follow-up period. Recrudescences may occur with all *Plasmodium* species when blood-stage parasites are not completely eliminated and they subsequently increase in numbers after the decline of drug levels in the blood [52]; they could therefore be an indication of emerging drug-resistant parasites. These recrudescences were treated successfully with either a second-line medicine or a repeat of the same medicine as for the primary infection on the basis of reports on therapeutic efficacy in the country of origin. Current information on therapeutic efficacy of antimalarials globally is, therefore, an important resource for the clinical management of imported malaria [53]. Furthermore, recrudescences, unless detected early by regular follow-up of patients for at least 28 days after treatment, could give rise to onward transmission of parasites and increase the risk of malaria re-establishment.

Transmission to mosquito vectors from imported malaria patients depend on the presence of gametocytes, the infectious stages of *Plasmodium* in the peripheral blood. In this study, only 19% of *P. falciparum* patients had detectable circulating gametocytes at the time of detection in contrast to the other three species in which these stages were present in 84% of patients. These differences have a biological basis in that *P. falciparum* immature gametocytes sequester in deep tissues and do not emerge into the peripheral circulation for at least 10 days after the onset of an infection, whereas in all other *Plasmodium* species, they mature and appear in the peripheral circulation within 2**–**3 days of a blood infection [54]. The risk of re-establishment of malaria may, therefore, be higher with *P. vivax* infections than with *P. falciparum* because more of them would have been infectious to the mosquito vector at the time of detection.

Profiling imported malaria has allowed the identification of high-risk populations, their travel destinations, health-seeking patterns, and use of preventive measures. These data have contributed to improving strategies to prevent the re-establishment of malaria. Many countries which have eliminated malaria in the past two decades are in the tropical zone and therefore have a high presence of malaria vectors [1]. Imported malaria places such countries under a continuous threat of the disease being re-established. Monitoring the time taken to diagnose imported malaria as an indicator in elimination and prevention of re-establishment programs will be important to reduce the mortality of travelers’ malaria and also the risk of malaria re-establishment. Health systems must take account of imported malaria now being largely associated with labor-related travel. Therefore, provision of healthcare to such vulnerable groups as travelers is important ethically, and as a health security measure.

## Conclusions

Over half of imported malaria patients were work-related travelers; therefore, providing information on travel risks and good access to affordable healthcare for travelers of all social and economic strata becomes an important component of travel health. Imported malaria reflects transmission intensities and trends in the countries of origin. Epidemiological features of imported malaria are specific to *Plasmodium* species and differences between them relate to aspects of their biology. Imported *P. ovale* infections manifest clinically much later than do other Plasmodia species. The risk of onward transmission from *P. falciparum* malaria is lower than that of other Plasmodia species because very few infections present with gametocytes if they are diagnosed early. Delays in diagnosis of imported malaria are largely on the part of the health system rather than due to patients’ hesitancy in seeking treatment. Time to diagnosis should be considered as a key indicator for monitoring the performance of prevention of malaria re-establishment programs. Continuous efforts are required to increase the awareness of imported malaria among travelers and healthcare professionals to reduce morbidity and mortality of travelers’ malaria, and to reduce the risk of re-establishing malaria in tropical countries after it has been eliminated.

## Supplementary Information

Supplementary Material 1: Table S1: STROBE checklist.

Supplementary Material 2: Table S2 and Table S3.

## Data Availability

The datasets generated and/or analyzed in this publication are not publicly available due to the fact that the patient database is the property of the Ministry of Health, Sri Lanka. In accordance with the official data governance and confidentiality policies of the Ministry of Health, we do not have permission to place it in a public repository or share it externally, apart from the manner in which we have provided the data in the tables, figures, and supplementary tables. All datasets on which the conclusions of the paper rely are provided in an anonymized form within the manuscript and its supporting files. Some of this data is provided in an aggregated form. Clarifications regarding data can be made through Dr. Champa Aluthweera, Director of the Anti Malaria Campaign, Sri Lanka, who is an author of this publication (email: champaaluthweera@gmail.com).

## Abbreviations

ACT: Artemisinin combination therapy
AL: Artemether-lumefantrine
AMC: Anti Malaria Campaign
DHA-PQ: Dihyro artemisinin piperquine
Hb: Hemoglobin
MoHNIM: Ministry of Health, Nutrition, and Indigenous Medicine
P: *Plasmodium*
PCR: Polymerase chain reaction
PHLT: Public health laboratory technician
RDT: Rapid diagnostic test
USD: United States dollar
WHO: World Health Organization

## References

[1] WHO. Countries and territories certified malaria free by WHO. Geneva: World Health Organisation; 2025. https://www.who.int/teams/global-malaria-programme/elimination/countries-and-territories-certified-malaria-free-by-who. Accessed 25 April 2025.

[2] Mischlinger J, Rönnberg C, Álvarez-Martínez MJ, Bühler S, Paul M, Schlagenhauf P, et al. Imported malaria in countries where malaria is not endemic: a comparison of semi-immune and nonimmune Travelers. Clin Microbiol Rev. 2020; 11;33(2):e00104–19. 10.1128/CMR.00104-19 PMC7067581 32161068

[3] Dupré A, Argy N, Houze S, Leleu A, Choquet C, Matheron S, et al. Imported malaria in metropolitan France, from recommendations to clinical practice – proposal for improvement. Infectious Diseases Now. 2021;51:667–72. 34464757 10.1016/j.idnow.2021.08.002

[4] Liu Y, Hsiang MS, Zhou H, Wang W, Cao Y, Gosling RD, et al. Malaria in overseas labourers returning to China: an analysis of imported malaria in Jiangsu Province, 2001–2011. Malar J. 2014;13:29. 24460982 10.1186/1475-2875-13-29 PMC3922785

[5] Li Z, Yang Y, Xiao N, Zhou S, Lin K, Wang D, et al. Malaria imported from Ghana by returning gold miners, China, 2013. Emerg Infect Dis. 2015;21:864–7. 25897805 10.3201/eid2105.141712 PMC4412230

[6] Lai S, Wardrop NA, Huang Z, Bosco C, Sun J, Bird T, et al. *Plasmodium falciparum* malaria importation from Africa to China and its mortality: an analysis of driving factors. Sci Rep. 2016;6:39524. 28000753 10.1038/srep39524 PMC5175130

[7] Zhou S, Li Z, Cotter C, Zheng C, Zhang Q, Li H, et al. Trends of imported malaria in China 2010–2014: analysis of surveillance data. Malar J. 2016;15:39. 26809828 10.1186/s12936-016-1093-0 PMC4727325

[8] Mendis K. Eliminating malaria should not be the end of vigilance. Nature. 2019;573:7. 31485061 10.1038/d41586-019-02598-1

[9] Anti Malaria Campaign, Sri Lanka. National Strategic Plan for prevention of re-establishment of malaria in Sri Lanka 2023–2027. https://apmen.org/resources/sri-lanka-national-strategic-plan-prevention-re-introduction-malaria-2023-2027. Accessed 25 April 2025

[10] von Elm E, Altman DG, Egger M, Pocock SJ, Gøtzsche PC, Vandenbroucke JP. STROBE Initiative. The Strengthening the Reporting of Observational Studies in Epidemiology (STROBE) Statement: guidelines for reporting observational studies. PLoS Med. 2007; 4(10):e296. 10.1371/journal.pmed.0040296 PMC2020495 17941714

[11] Datta R, Mendis K, Wikremasinghe R, Premaratne R, Fernando D, Parry J, et al. Role of a dedicated support group in retaining malaria-free status of Sri Lanka. J Vector Borne Dis. 2019;56:66–9. 31070169 10.4103/0972-9062.257778

[12] Gunasekera WMKT, Premaratne R, Fernando D, Munaz M, Piyasena MGY, Perera D, et al. A comparative analysis of the outcome of malaria case surveillance strategies in Sri Lanka in the prevention of re-establishment phase. Malar J. 2021;20:80. 33563273 10.1186/s12936-021-03621-5 PMC7871399

[13] Standard Operating Procedures for the activities performed in laboratories providing malaria microscopy services, AMC 2014. http://www.malariacampaign.gov.lk/images/Publication%20Repository/SOP/SOPMM.pdf. Accessed 26 August 2025.

[14] WHO. Management of severe malaria: a practical handbook, 3rd edn. Geneva: World Health Organization; 2012.

[15] WHO. Guidelines for malaria. Geneva: World Health Organization; 2022. (WHO/UCN/GMP/2022.01Rev.2). https://www.who.int/publications/i/item/guidelines-for-malaria. Accessed 9 April 2025.

[16] Karunasena VM, Marasinghe M, Amarasinghe S, Koo C, Senaratne PAS, Hasantha MBR, et al. The first introduced malaria case reported from Sri Lanka after elimination: implications for preventing the re-introduction of malaria in recently eliminated countries. Malar J. 2019;18:210. 31234941 10.1186/s12936-019-2843-6 PMC6591994

[17] Anti Malaria Campaign. The manual for parasitological surveillance in prevention of reintroduction / re-establishment of Malaria in Sri Lanka. Office of the Director General of Health Services, Ministry of Health and Indigenous Medical Services, Sri Lanka; 2019. http://www.malariacampaign.gov.lk/images/PublicNotice-Repository/Manual-for-Parasitological-Surveillance-in-prevention-of-reintroduction--or-reestablishment-of-malaria-in-Sri-Lanka.pdf. Accessed 9 April 2025.

[18] Anti Malaria Campaign, Ministry of Health, Sri Lanka. Guidelines for the management and treatment of patients with malaria. Third Edition, 2023. http://www.malariacampaign.gov.lk/images/publication_repository/Guidelines/Treatment-guidelines-Third-Edition--2023.pdf. Accessed 9 April 2025.

[19] Seneviratne S, Fernando D, Chulasiri P, Gunasekera K, Thenuwara N, Aluthweera C, et al. A malaria death due to an imported *Plasmodium falciparum* infection in Sri Lanka during the prevention of re-establishment phase of malaria. Malar J. 2023;22:243. 37620890 10.1186/s12936-023-04681-5 PMC10463374

[20] Anti Malaria Campaign, Ministry of Health, Sri Lanka. Guidelines on malaria chemotherapy and management of patients with malaria. 2014. General Circular No: 02–112/2014. http://amc.health.gov.lk/Circulars/Treatment-guidelines_Malaria.pdf. Accessed 9 April 2025.

[21] WHO. Towards a malaria free world: elimination of malaria and prevention of re-establishment in Sri Lanka. Geneva: World Health Organization, 2023. https://iris.who.int/bitstream/handle/10665/376553/9789240087026-eng.pdf?sequence. Accessed 25 April 2025.

[22] Premaratne R, Wickremasinghe R, Ranaweera D, Gunasekera W, Hevawitharana M, Pieris L, et al. Technical and operational underpinnings of malaria elimination from Sri Lanka. Malar J. 2019;18:256. 31358007 10.1186/s12936-019-2886-8 PMC6664748

[23] Chulasiri P, Ranaweera P, Sudarshan P, Jayasinghe M, Harishchandra J, Gunasekera K, et al. Transfusion-induced *Plasmodium falciparum* malaria in a beta thalassaemia patient during the prevention of re-establishment phase in Sri Lanka. Malar J. 2021;20:352. 34445999 10.1186/s12936-021-03881-1 PMC8390059

[24] Gunasekera KT, Premaratne RG, Handunnetti SM, Weerasena J, Premawansa S, Fernando D. Msp1, msp2 and glurp genotyping to differentiate *Plasmodium falciparum* recrudescence from reinfections during prevention of re-establishment phase, Sri Lanka, 2014–2019. Malar J. 2024;23:35. 38281044 10.1186/s12936-024-04858-6 PMC10821543

[25] Anti Malaria Campaign, Sri Lanka. Malaria prophylaxis for travellers: guidelines for healthcare workers. 2019. http://www.malariacampaign.gov.lk/images/PublicNotice-Repository/Malaria-Prophylaxis-for-Travellers-Guideline-for-Healthcare-Workers--AMC-2019.pdf. Accessed 10 April 2025.

[26] Ranaweera AD, Danansuriya MN, Pahalagedera K, Gunasekera WMKT de AW, Dharmawardena P, Mak KW, et al. Diagnostic challenges and case management of the first imported case of *Plasmodium knowlesi* in Sri Lanka. Malar J. 2017; 16:126. 10.1186/s12936-017-1776-1 PMC5361730 28327145

[27] WHO. World Malaria Report 2023. Geneva: World Health Organization, 2023. https://www.who.int/publications/i/item/9789240086173. Accessed 10 April 2025

[28] Li X, Deng L, Yang H, Wang H. Effect of socioeconomic status on the healthcare-seeking behaviour of migrant workers in China. PLoS ONE. 2020;15(8):e0237867. 32813702 10.1371/journal.pone.0237867 PMC7444513

[29] Fernando SD, Rodrigo R, Silva N de Silva, Semege S, Rajapakse S, Samaranayake N, et al. Educating the security forces a high risk group in malaria elimination efforts; an example from Sri Lanka. *Int Health.* 2014; 6:196–202. 10.1093/inthealth/ihu044 25061075

[30] Fernando SD, Dharmawardana P, Semege S, Epasinghe G, Senanayake N, Rodrigo C, et al. The risk of imported malaria in security forces personnel returning from overseas missions in the context of prevention of re-introduction of malaria to Sri Lanka. Malar J. 2016;15:144. 26955813 10.1186/s12936-016-1204-y PMC4784464

[31] Fernando SD, Booso R, Dharmawardena P, Harintheran AA, Raviraj K, Rodrigo C, et al. The need for preventive and curative services for malaria when military is deployed in endemic overseas territories: a case study and lessons learnt. Mil Med Res. 2017;4:19. 28593051 10.1186/s40779-017-0128-3 PMC5460350

[32] Ministry of Health, Sri Lanka. Sri Lanka National Migration Health Policy, Cabinet Memorandum No 11/2140/509/159 dated 14^th^ Nov 2014. Available from: http://www.previousmoh.health.gov.lk/moh_final/english/public/elfinder/files/publications/publishpolicy/10_Migration%20Health.pdf. Accessed 9 April 2025.

[33] Ranaweera P, Wickremasinghe R, Mendis K. Preventing the re-establishment of malaria in Sri Lanka amidst the COVID-19 pandemic. Malaria J. 2020;19:386. 10.1186/s12936-020-03465-5 PMC7605332 33138814

[34] Camargo M, Soto-De León SC, Del Río-Ospina L, Páez AC, González Z, González E, et al. Micro-epidemiology of mixed-species malaria infections in a rural population living in the Colombian Amazon region. Sci Rep. 2018;8:5543. 29615693 10.1038/s41598-018-23801-9 PMC5883018

[35] Sitali L, Miller JM, Mwenda MC, Bridges DJ, Hawela MB, Hamainza B, et al. Distribution of *Plasmodium* species and assessment of performance of diagnostic tools used during a malaria survey in southern and western provinces of Zambia. Malar J. 2019;18:130. 30971231 10.1186/s12936-019-2766-2 PMC6458729

[36] Guerra RI, Ore M, Valdivia HO, Bishop DK, Ramos M, Mores CN, et al. A cluster of the first reported *Plasmodium ovale* spp. infections in Peru occurring among returning UN peace-keepers, a review of epidemiology, prevention and diagnostic challenges in nonendemic regions. Malar J. 2019;18:176. 31113437 10.1186/s12936-019-2809-8 PMC6530030

[37] Woldearegai TG, Lalremruata A, Nguyen TT, Gmeiner M, Veletzky L, Tazemda-Kuitsouc GB, et al. Characterization of *Plasmodium* infections among inhabitants of rural areas in Gabon. Sci Rep. 2019;9:9784. 31278305 10.1038/s41598-019-46194-9 PMC6611864

[38] Zhang T, Lyu X, Xu X, Wang S, Jiang J, Liu Z, et al. The rising proportion of *Plasmodium ovale* spp. in imported malaria in Anhui Province, China: a retrospective propensity score-matched case-control study. Acta Trop. 2025;264:107573. 40049310 10.1016/j.actatropica.2025.107573

[39] Warrell DA. Clinical features of malaria. In: Warrell DA, Gilles HM, editors. Essential malariology. New York: Oxford University Press; 2002. p. 192–4.

[40] Trampuz A, Jereb M, Muzlovic I, Prabhu RM. Clinical review: severe malaria. Crit Care. 2003;7:315–23. 12930555 10.1186/cc2183 PMC270697

[41] Nabarro LEB, Nolder D, Broderick C, Nadjm B, Smith V, Blaze M, et al. Geographical and temporal trends and seasonal relapse in *Plasmodium ovale* spp. and *Plasmodium malariae* infections imported to the UK between 1987 and 2015. BMC Med. 2018;16:218. 30477484 10.1186/s12916-018-1204-6 PMC6260574

[42] Wångdahl A, Sondén K, Wyss K, Stenström C, Björklund D, Zhang J, et al. Relapse of *Plasmodium vivax* and *Plasmodium ovale* malaria with and without primaquine treatment in a nonendemic area. Clin Infect Dis. 2002;74:1199–207. 10.1093/cid/ciab610 PMC8994585 34216464

[43] Goff ML, Kendjo E, Thellier M, Piarroux R, Boelle PY, Jauréguiberry S. Impact of chemoprophylaxis on *Plasmodium vivax* and *Plasmodium ovale* infection among civilian travelers: a nested case-control study with a counterfactual approach on 862 patients. Clin Infect Dis. 2023;76(3):e884–93. 35962785 10.1093/cid/ciac641

[44] Seneviratne S, Fernando D, Wickremasinghe R, Senarathne S, Chulasiri P, Thenuwara N, et al. An epidemiological analysis of severe imported malaria infections in Sri Lanka, after malaria elimination. Malar J. 2024;23:195. 38909255 10.1186/s12936-024-05014-w PMC11193279

[45] Anti Malaria Campaign, Sri Lanka. Advice to travellers. http://amc.health.gov.lk/en/travelers-guide. Accessed 25 April 2025.

[46] Anti Malaria Campaign, Sri Lanka. PROMIS initiative. https://www.facebook.com/Antimalariacampaign.SL?comment_id=Y29tbWVudDoxNDg4ODI5ODEwODcwMDJfMjA5MTA1MDI2NDM4OTU0OQ%3D%3D. Accessed 25 April 2025.

[47] Wickremasinghe AR. Elimination of malaria from Sri Lanka and beyond; lessons for other countries in elimination phase. Ceylon Med J. 2023;68:si 39–45. 37610920

[48] Dharmawardena P, Premaratne R, Mendis K, Wickremasinghe R, Rodrigo C, Harintheran A, et al. Effectiveness of passive case detection for imported malaria in a hospital setting in Sri Lanka during the prevention of re-introduction phase of malaria. Int Health. 2019;11:64–70. 30137418 10.1093/inthealth/ihy061

[49] Gallien S, Taieb F, Schlemmer F, Lagrange-Xelot M, Atlan A, Sarfati C, et al. Failure of atovaquone/proguanil to prevent *Plasmodium ovale* malaria in traveler returning from Cameroon. Travel Med Infect Dis. 2008;6:128–9. 18486067 10.1016/j.tmaid.2008.01.011

[50] Rojo-Marcos G, Rubio-Muñoz JM, Angheben A, et al. Prospective comparative multi-centre study on imported *Plasmodium ovale wallikeri* and *Plasmodium ovale curtisi*infections. Malar J. 2018;17:399. 30376868 10.1186/s12936-018-2544-6 PMC6208040

[51] Nolder D, Oguike MC, Maxwell-Scott H, Niyazi HA, Smith V, Chiodini PL, et al. An observational study of malaria in British travellers: *Plasmodium ovale wallikeri* and *Plasmodium ovale curtisi* differ significantly in the duration of latency. BMJ Open. 2013;3(5):e002711. 23793668 10.1136/bmjopen-2013-002711 PMC3657643

[52] Richter J, Franken G, Holtfreter MC, Walter S, Labisch A, Mehlhorn H. Clinical implications of a gradual dormancy concept in malaria. Parasitol Res. 2016;115:2139–48. 27079460 10.1007/s00436-016-5043-0

[53] WHO. Malaria threat maps. Antimalarial drug efficiency and resistance. Geneva: World Health Organisation; 2025. https://apps.who.int/malaria/maps/threats/ Accessed 30 July 2025.

[54] Garnham PCC. Malaria parasites and other Haemosporidia. 1966, xviii + 1114 pp. Blackwell Scientific Publications Ltd. Oxford, UK.

