# Supplementary material for: The profile of imported malaria in Sri Lanka from 2013 to 2023
Source: BMC Glob Public Health. 2026 Jan 14;4:8. doi: 10.1186/s44263-026-00242-5 (PMC12805712; doi:10.1186/s44263-026-00242-5)
Supplement: Supplementary file 2 — Supplementary Material 2: Table S2 and Table S3. [file 44263_2026_242_MOESM2_ESM.docx]

**Table S2. Imported malaria infections diagnosed in Sri Lank, by year and *Plasmodium* species**

| **Year** | **Total number of imported malaria** | ***P. ovale***  ***Number (%)*** | ***P. malariae***  ***Number (%)*** | ***P. falciparum***  ***Number (%)*** | ***P. vivax***  ***Number (%)*** |
| --- | --- | --- | --- | --- | --- |
| 2013 | 95 | 1 (1.1) | 0 | 42 (44.2) | 52 (54.7) |
| 2014 | 49 | 0 | 1 (2.0) | 20 (40.8) | 28 (57.1) |
| 2015 | 36 | 2 (5.6 ) | 0 | 17 (47.2) | 17 (47.2) |
| 2016 | 41 @ | 5 (12.2) | 1 (2.4) | 18 (43.9) | 17 (41.5)^@^ |
| 2017 | 57 | 3 (5.3) | 01 (1.8) | 26 (45.6) | 27 (47.4) |
| 2018 | 47 | 3 (6.4) | 0 | 15 (31.9) | 29 (61.7) |
| 2019 | 53 | 3 (5.7) | 2 (3.8) | 24 (45.3) | 24 (45.3) |
| 2020 | 30* | 8 (26.7) | 2 (6. 7) | 9* (30.0) | 11 (36.7) |
| 2021 | 25 | 7 ( 28.0) | 1 (4.0) | 12 (48.0) | 5 (20.0) |
| 2022 | 37 | 7 (18.9) | 1 (2.7) | 27 (73.0) | 2 (5.4) |
| 2023 | 62** | 11 (17.7 ) | 1 (1.6) | 46 (74.2)** | 4 (6.5) |
| **TOTAL** | 532 | 50 (9.4) | 10 (1.9) | 256 (48.3) | 216 (40.6)^@^ |
| **Total from Africa** | 322 | 48 | 10 | 231 | 33 |
| **Total from India** | 155 | 1 | 0 | 14 | 140 |
| **Total from other countries** | 55 | 1 | 0 | 11 | 42 |

@ Includes one *P. knowlesi* case in the *P. vivax* cell

*Includes a mixed infection of *P. falciparum* and *P. ovale*

******Includes one mixed infection of *P. falciparum* and *P. malariae*

**Table S3. Characteristics of imported malaria cases in Sri Lanka from 2013-2023 by *Plasmodium* species, gender and age group.**

| **Characteristic** | **Total number of imported malaria infections** | ***P. ovale***  ***Number (%)*** | ***P. malariae***  ***Number (%)*** | ***P. falciparum***  ***Number (%)*** | ***P. vivax***  ***Number (%)*** | ***Other****  ***Number (%)*** |
| --- | --- | --- | --- | --- | --- | --- |
| **Gender** | | | | | | |
| Male | 479 | 47 (9.8) | 9 (1.9) | 240 (50.1) | 180 (37.6) | 3 (0.6) |
| Female | 53 | 3 (5.7) | 1 (1.9) | 14 (26.4) | 35 (66.0) | 0 |
| **Total** | 532 | 50 (9.4) | 10 (1.9) | 254 (47.7) | 215 (40.4) | 3 (0.6) |
| **Age** | | | | | | |
| 0-10 | 10 | 0 | 0 | 1 (10.0) | 9 (90.0) | 0 |
| 11-20 | 20 | 0 | 1 (5.0) | 8 (40.0) | 11 (55.0) | 0 |
| 21-30 | 141 | 9 (6.4) | 3 (2.1) | 69 (48.9) | 60 (42.6) | 0 |
| 31-40 | 163 | 21 (12.9) | 5 (3.1) | 80 (49.1) | 55 (33.7) | 2 (1.2) |
| 41-50 | 125 | 15(12.0) | 1 (0.8) | 65 (52.0) | 43 (34.4) | 1 (0.8) |
| 51-60 | 50 | 3 (6.0) | 0 | 26 (52.0) | 21 (42.0) | 0 |
| 61-70 | 21 | 2 (9.5) | 0 | 5 (23.8) | 14 (42.0) | 0 |
| 71-80 | 2 | 0 | 0 | 0 | 2 (66.7) | 0 |
| **Total** | 532 | 50 (9.4) | 10 (1.9) | 254 (47.7) | 215 (40.4) | 3 (0.6) |

** includes two mixed infections and one P. knowlesi* infection
